# High Amylose Resistant Starch Diet Ameliorates Oxidative Stress, Inflammation, and Progression of Chronic Kidney Disease

**DOI:** 10.1371/journal.pone.0114881

**Published:** 2014-12-09

**Authors:** Nosratola D. Vaziri, Shu-Man Liu, Wei Ling Lau, Mahyar Khazaeli, Sohrab Nazertehrani, Seyed H. Farzaneh, Dorothy A. Kieffer, Sean H. Adams, Roy J. Martin

**Affiliations:** 1 Division of Nephrology, University of California Irvine, Irvine, California, United States of America; 2 Graduate Group in Nutritional Biology and Department of Nutrition, University of California Davis, Sacramento, California, United States of America; 3 Obesity & Metabolism Research Unit, USDA-ARS Western Human Nutrition Research Center, Davis, California, United States of America; Emory University, United States of America

## Abstract

Inflammation is a major mediator of CKD progression and is partly driven by altered gut microbiome and intestinal barrier disruption, events which are caused by: urea influx in the intestine resulting in dominance of urease-possessing bacteria; disruption of epithelial barrier by urea-derived ammonia leading to endotoxemia and bacterial translocation; and restriction of potassium-rich fruits and vegetables which are common sources of fermentable fiber. Restriction of these foods leads to depletion of bacteria that convert indigestible carbohydrates to short chain fatty acids which are important nutrients for colonocytes and regulatory T lymphocytes. We hypothesized that a high resistant starch diet attenuates CKD progression. Male Sprague Dawley rats were fed a chow containing 0.7% adenine for 2 weeks to induce CKD. Rats were then fed diets supplemented with amylopectin (low-fiber control) or high fermentable fiber (amylose maize resistant starch, HAM-RS2) for 3 weeks. CKD rats consuming low fiber diet exhibited reduced creatinine clearance, interstitial fibrosis, inflammation, tubular damage, activation of NF_k_B, upregulation of pro-inflammatory, pro-oxidant, and pro-fibrotic molecules; impaired Nrf2 activity, down-regulation of antioxidant enzymes, and disruption of colonic epithelial tight junction. The high resistant starch diet significantly attenuated these abnormalities. Thus high resistant starch diet retards CKD progression and attenuates oxidative stress and inflammation in rats. Future studies are needed to explore the impact of HAM-RS2 in CKD patients.

## Introduction

Systemic inflammation and oxidative stress play central roles in progression of chronic kidney disease (CKD) and the associated complications. There is mounting evidence pointing to marked alteration of the structure and function of the gut microbiome and its role in the pathogenesis of CKD-associated systemic inflammation. Recent studies have revealed that advanced CKD results in disruption of the gastro-intestinal epithelial barrier structure and function [1]–[3] and profound changes in the composition and function of the intestinal microbiota [4], [5], events that contribute to the pathogenesis of systemic inflammation. Several factors contribute to the CKD-associated impairment of the gut epithelial barrier structure and function and alteration of intestinal microbiota. Chief among them is the rise in urea concentration in the body fluids which leads to its heavy influx into the gastrointestinal tract, where it accommodates the dominance of urease-possessing bacteria, conversion of urea to ammonia [NH_2_-CO-NH_2_+H_2_O→ 2NH_3_+CO_2_] and formation of ammonium hydroxide [NH_3_+H_2_O →NH_4_(OH)]. These caustic products, in turn, damage the tight junction proteins that face the lumen and seal the gap between the epithelial cells [6], [7]. The resulting disruption of the epithelial barrier promotes inflammation by facilitating the paracellular translocation of endotoxin, noxious microbial and other waste products from the lumen to the intestinal wall and systemic circulation [8]–[11]. The other likely cause of the altered intestinal microbiome is dietary restriction of potassium-rich fruits and vegetables (to prevent hyperkalemia) which are common sources of soluble and insoluble dietary fiber. These indigestible complex carbohydrates are the primary source of nutrients for the symbiotic intestinal bacteria that convert the dietary fiber to short chain fatty acids (SCFA). SCFA produced by the intestinal microbiota are vital nutrients for the colonic epithelial cells and the regulatory T lymphocytes (T-reg) that are essential for the maintenance of the immunological self-tolerance and limitation of the inflammatory response [12]. Reduced intake of fermentable dietary fiber may, therefore, contribute to the CKD-associated changes in the structure/function of the gut microbiota by limiting the population of SCFA-forming bacteria and to systemic inflammation by lowering the population and function of T-reg. In fact, the T-reg cell population is markedly reduced in end-stage renal disease (ESRD) patients [13], [14] and recent studies have shown marked reduction of SCFA-producing bacterial families in these patients [5].

Given the importance of dietary fiber in preservation of symbiotic microbiota and production of SCFA, restriction of potassium-rich fruits and vegetables can potentially contribute to the systemic inflammation and progression of CKD in this population. The present study was designed to test the hypothesis that consumption of a diet high in resistant starch [high amylose maize resistant starch type 2 (HAM-RS2)] attenuates oxidative stress, inflammation, and progression in a rodent model of CKD.

## Methods

### Study groups

Male Sprague-Dawley rats weighing 200–220 g (Harlan Sprague Dawley Inc, Indianapolis, IN) were fed a powdered chow containing 0.7% adenine for 2 weeks to induce chronic interstitial nephropathy. They were then randomized to receive a pelleted low-fiber control diet containing highly-digestible starch (amylopectin) (n = 9) or a high-fiber diet containing 59% high amylose maize resistant starch (HI-MAIZE 260) (n = 9) for 3 weeks. The purified pelleted diets were prepared by Harlan Laboratories (Madison WI) using the following ingredients (g/kg): casein (140), maltodextrin (149), soybean oil (70), mineral mix, AIN-93M-MX (94049) (35) Vitamin Mix, AIN-93-VX (94047) (10), Choline Bitartrate (2.5), TBHQ, antioxidant (0.014). The control diet (TD.130689) contained AMIOCA Waxy Maize Starch (462 g/kg) plus cellulose (128 g/kg), whereas the Resistant Starch Diet (TD.130688) contained Hi-Maize 260 resistant starch (590 g/kg). Diets were isocaloric (3.4 kcal/g) with energy content of 14.5%/66.9%/18.6% protein/carbohydrate/fat respectively. A third group of rats consuming a regular chow (Harlan Laboratories) served as healthy controls (n = 6). The animals were provided free access to food instead of being pair fed. This is because uremia is invariably associated with anorexia and reduced food intake. Thus pair feeding would have caused malnutrition in the healthy rats which would have disqualified them as true normal controls and would have diminished the translational value of the study. The animals were placed in metabolic cages for a 24-h urine collection and arterial pressure was measured by tail plethysmography as described previously [15]. They were then anesthetized (Ketamine/Xylazine IP) and euthanized by cardiac exsanguination between the hours of 8–11 AM. The kidneys were immediately removed and processed for histological evaluation and Western blot analyses. In addition ascending colon was removed and processed for determination of the epithelial tight junction proteins by Western blot analysis. The water content of the stools harvested from the descending colon was quantified using freeze drying technique. Plasma creatinine and urea concentrations and urinary protein excretion were measured using the following kits: QuantiChrom TM Creatinine Assay Kit, Cat # DICT-500, QuantiChrom TM Urea Assay Kit, Cat # DIUR-500 (BioAssay Systems, Hayward, CA) and Rat Urinary Protein Assay Kit, Cat # 9040 (Chondrex Inc. Redmond, WA). All experiments were approved by the University of California Irvine Institutional Committee for the Use and Care of Experimental Animals.

### Histologic Analysis

Cross-sections of the kidneys were fixed in 10% buffered formalin and two micron paraffin embedded sections were cut and stained with hematoxylin & eosin (H&E) or periodic acid Schiff (PAS). The slides were examined under light microscopy by a pathologist in a blinded manner. Histologic findings were graded according to the modified Banff classification criteria in 20 randomly selected non-overlapping fields per rat H.E. and PAS stained kidney tissues.

### Western blot analyses

Cytoplasmic and nuclear extracts of the renal tissue were prepared as described previously [16]. The proteins of interest in the cytoplasmic and/or nuclear fractions of the kidney tissue were measured by Western blot analysis as previously described [17], [18] using antibodies against rat NF-κB p65, MCP-1, inducible nitric oxide synthase (iNOS), NOX4, P47^phox^, nitrotyrosine, Nrf2, glutathione peroxidase (GPX), CuZn-SOD, cyclooxygenase-1 (COX-1), cyclooxygenase-2 (COX-2), heme oxygenase-1 (HO-1), catalase, and gp91^phox^. Antibodies to claudin-1 and occludin were used to examine colonic epithelial tight junction in the study animals. Antibodies against histone H1 and GAPDH were used for measurements of housekeeping proteins for nuclear and cytosolic target proteins, respectively.

### Statistical analysis

Analysis of variance and multiple range tests were employed in statistical analysis of the data using GraphPad Prism 4 software (GraphPad Software, San Diego, CA). Data are presented as mean ± SEM. P values less than 0.05 were considered significant.

## Results

### General phenotype data

Data are shown in Table 1. Compared to the healthy control group, the CKD groups showed significant increases in serum urea and creatinine concentrations and significant reduction in creatinine clearance. This was associated with mild proteinuria, marked polyuria, and a significant reduction in urine specific gravity consistent with the presence of severe tubulointerstitial nephropathy. The severity of these abnormalities was significantly less in CKD rats fed HAM-RS2 diet compared to CKD rats consuming low fiber control diet. The body weight in the CKD rats consuming HAM-RS2 diet was similar to those fed the low-fiber control diet. Both CKD groups weighed significantly less than the healthy control group. No significant difference was found in the water content of stools between the CKD rats fed low-fiber diet (8.6±0.07%) and healthy control animals (7.3±0.06%). However, the stool water content was significantly increased in CKD rats consuming HAM-RS2 diet (28.5±0.04%, P<0.001).

**Table 1:** Phenotype data in the control (CTL), and CKD rats fed low fiber starch diet (CKD), and resistant starch supplemented diet (CKD-RS) groups in rats.

	CTL (n = 6)	CKD (n = 9)	CKD-RS (n = 9)
**Body weight (g)**	376±7	343±2*	349±7*
**Serum creatinine (mg/dl)**	0.36±0.05	0.88±0.05*	0.76±0.09* **^#^**
**Serum urea (mg/dl)**	34.18±1.8	73.9±4.8*	69.4±7*
**Urine volume (ml/24 h)**	10.7±1	35.3±2.5*	20.1±2.6* **^#^**
**CCr (ml/min/kg)**	7.07±0.51	1.97±0.21*	3.01±0.40* **^#^**
**Urine protein (mg/24 h)**	15.5±3.1	62.9±4.0*	34.5±2.7* **^#^**
**Urine specific gravity**	1.03±0.005	1.003±0.0005*	1.012±0.001* **^#^**

*p<0.05 compared to CTL, **^#^** *p*<0.05 compared to CKD.

### Histological data

**Figure 1:**
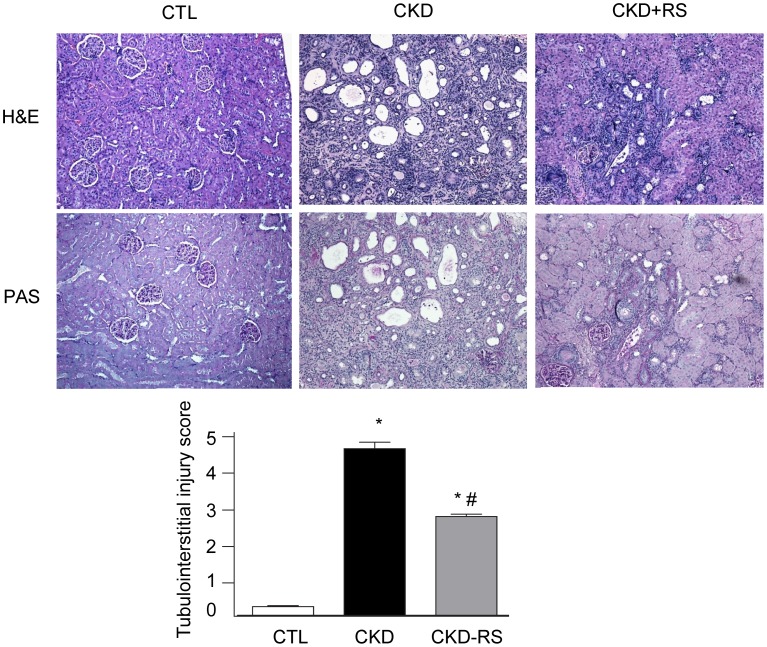
Representative photomicrographs of the PAS & H&E stained kidney sections in a normal control rat, a CKD rat fed low fiber, and a CKD rat fed high resistant starch diet [Magnification x10]. The kidney tissue in the CKD animals exhibited significant tubulo-interstitial injury and fibrosis and heavy inflammatory cell infiltration which were significantly improved with high resistant starch diet (Upper panel). Bar graphs depicting tubulointerstitial injury scores in the study groups (Lower panel).

Representative photomicrographs of PAS & H&E stained kidney sections are shown in Fig. 1. The kidney tissues in rats with adenine-induced CKD showed severe tubulointerstitial injury marked by tubular atrophy and dilation, severe interstitial inflammatory cell infiltration, and interstitial fibrosis. The severity of tubulo-interstitial injury in CKD rats fed HAM-RS2 diet was significantly less than that found in the CKD rats fed low-fiber control diet.

### Inflammatory, oxidative, and fibrosis pathway data

**Figure 2:**
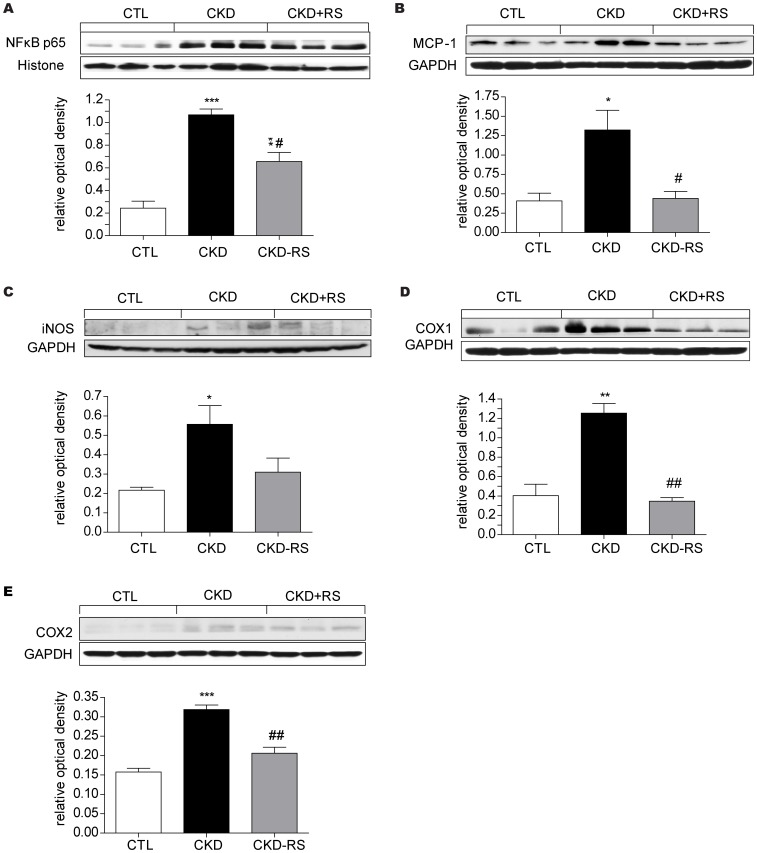
Representative Western blots and group data depicting nuclear content of p65 active subunit of NF-κB and protein abundance of MCP-1, iNOS, COX-1, and COX-2 in the renal tissues of the normal control rats (n = 6) and CKD rats fed low fiber (n = 9) or high resistant starch supplemented diets (n = 9). Data are means ± SE. *P<0.05, **P<0.01, ***P<0.001 VS CTL group, #P<0.05, ##P<0.01, ###P<0.001 VS CKD group.

**Figure 3:**
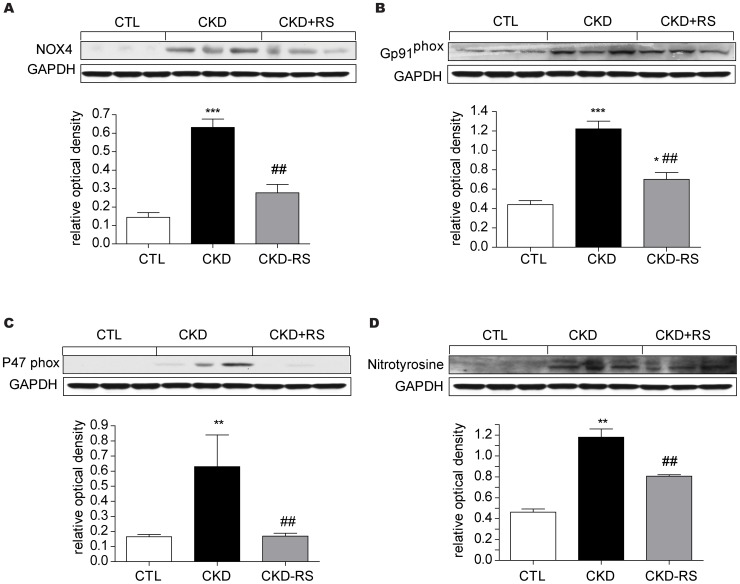
Representative Western blots and group data depicting the NAD(P)H oxidase subunits (NOX-4, gp91phox) and nitrotyrosine abundance in the renal tissues of the normal control rats (n = 6) and CKD rats fed low fiber (n = 9) or high resistant starch supplemented diets (n = 9). Data are means ± SE. *P<0.05, **P<0.01, ***P<0.001 VS CTL group, #P<0.05, ##P<0.01, ###P<0.001 VS CKD group.

**Figure 4:**
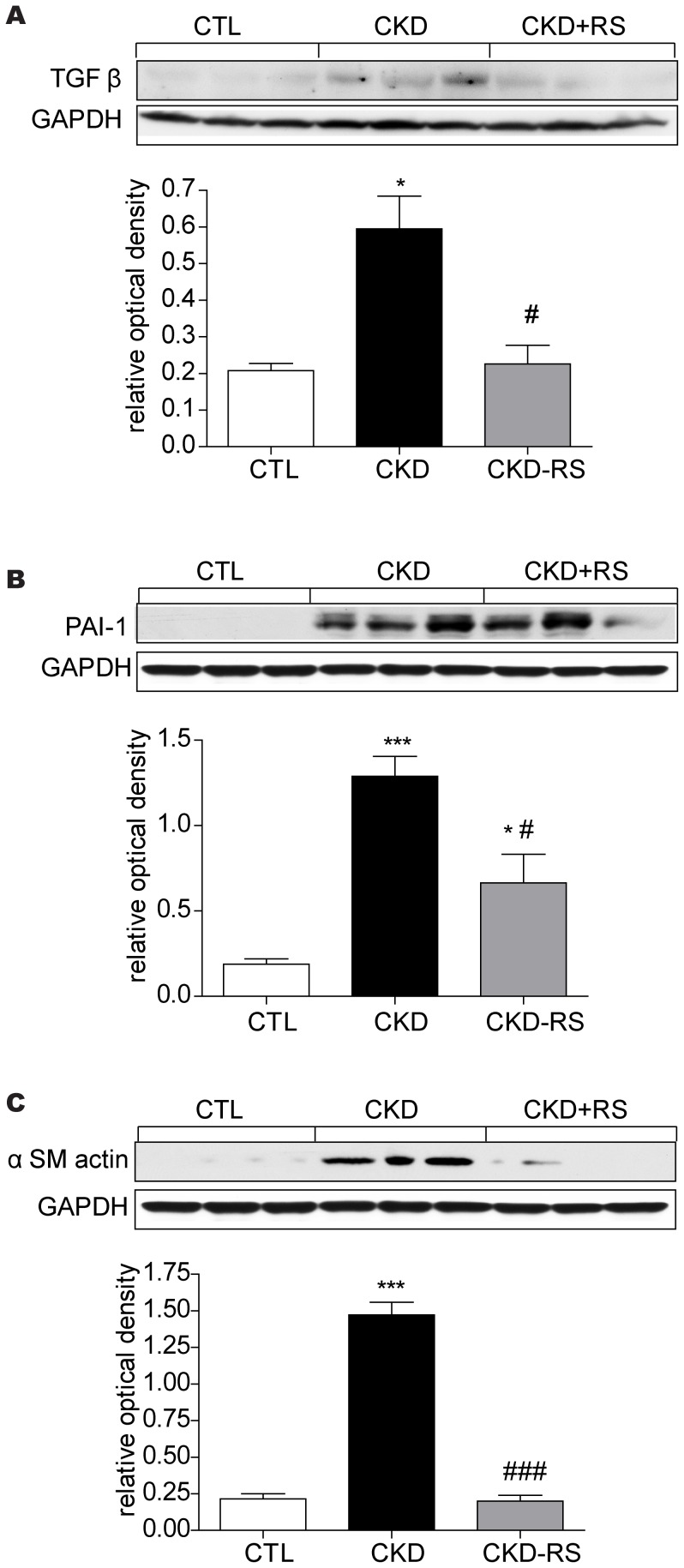
Representative Western blots and group data depicting TGF-β, α-SM actin, and PAI-1 abundance in the renal tissues of the normal control rats (n = 6) and CKD rats fed low fiber (n = 9) or high resistant starch supplemented diets (n = 9). Data are means ± SE. *P<0.05, **P<0.01, ***P<0.001 VS CTL group, #P<0.05, ##P<0.01, ###P<0.001 VS CKD group.

Data are shown in Figs. 2, 3 and 4. The kidney tissues from the CKD rats showed a marked increase in nuclear translocation of p65, pointing to activation of NFκB. This was accompanied by significant upregulation of pro-inflammatory and reactive oxygen species-generating molecules including MCP-1, iNOS, COX-1, COX-2, gp91^Phox^, NOX-4, and P47^Phox^. In confirmation of earlier studies [19] kidney tissues in the CKD rats showed marked accumulation of nitrotyrosine. This is a consequence of nitric oxide interaction with superoxide, leading to formation peroxynitrite (NO+O°2–> ONOO-), a highly reactive nitrogen species which avidly attacks and denatures lipids, nucleic acids, and proteins. Activation of inflammatory and oxidative pathways in the kidneys of CKD rats was accompanied by marked upregulation of TGF β, PAI-1, and α-SM actin, pointing to activation of fibrotic pathway. Consumption of the HAM-RS2 diet significantly attenuated all of the abovementioned abnormalities.

**Figure 5:**
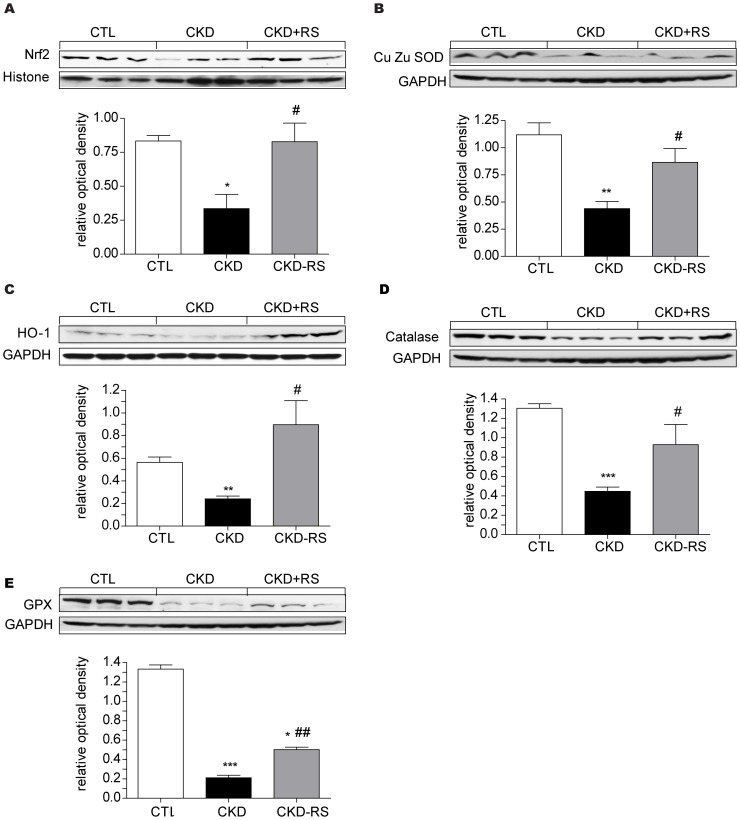
Representative Western blots and group data depicting nuclear translocation of Nrf2 and protein abundances of its downstream gene products, CuZn-SOD, catalase, heme oxygenase-1 (HO-1) and glutathione peroxidase (GPX) in the renal tissues of the normal control rats (n = 6) and CKD rats fed low fiber (n = 9) or resistant starch supplemented diets (n = 9). *P<0.05, **P<0.01, ***P<0.001 VS CTL group, #P<0.05, ##P<0.01, ###P<0.001 VS CKD group.

Nrf2 pathway data (Fig. 5) - The kidney tissues from the CKD rats showed marked reduction in nuclear translocation of Nrf2 and down-regulation of its key target gene products including CuZn-SOD, catalase, glutathione peroxidase, and heme oxygenase-1. These findings confirm the results of our earlier studies [17], [18] and point to impairment of the Nrf2 pathway and its contribution to the prevailing oxidative stress and inflammation in CKD. Consumption of the HAM-RS2 diet resulted in partial improvement of nuclear translocation of Nrf2 and expression of its measured target gene products.

### Colonic epithelial tight junction data (Fig. 6)

The colonic tissues from the CKD rats consuming low fiber diet showed marked reduction in claudin-1 and occludin levels confirming our earlier study [2]. Consumption of the resistant starch-fortified diet significantly increased claudin-1 and occludin levels to the levels approaching those found in the control animals.

**Figure 6:**
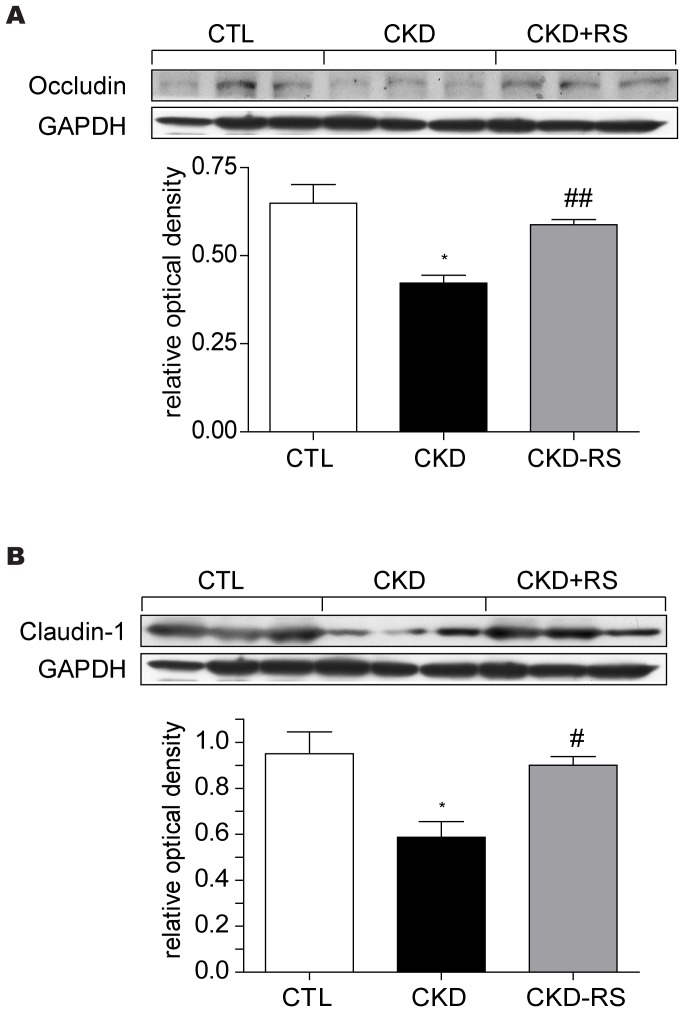
Representative Western blots and group data depicting protein abundances of claudin-1 and occludin in the ascending colons of the normal control rats (n = 6) and CKD rats fed low fiber (n = 9) or resistant starch supplemented diets (n = 9).

## Discussion

The diseased kidney tissues in rats with adenine-induced chronic tubulo-interstitial nephropathy employed in the present study exhibited marked upregulation of inflammatory, oxidative, and pro-fibrotic pathways that was coupled with the impaired activity of Nrf2 and down-regulation of its antioxidant and cytoprotective target gene products. These findings are consistent with the results of our earlier studies employing rats with CKD induced by 5/6 nephrectomy in which the prominent lesion is glomerulosclerosis [17], [18]. In confirmation of our earlier study [2] the CKD animals exhibited marked depletion of the colonic tissue claudin-1 and occludin which are the key components of the epithelial tight junction. Consumption of the fermentable dietary fiber, HAM-RS2, attenuated the severity of renal histological abnormalities including tubular damage, interstitial fibrosis, and inflammatory cell infiltration in this model. This was associated with significant improvement in renal function as evidenced by higher creatinine clearance, lower serum urea and creatinine concentrations, and significant attenuation of the urinary concentrating defect and associated polyuria. The observed improvements of the renal structure and function in the CKD rats consuming HAM-RS2 was accompanied by partial reversal of the upregulation of oxidative, inflammatory, and pro-fibrotic pathways, and partial restoration of Nrf2 activity and expression of its key target gene products. Amelioration of oxidative stress, inflammation, and fibrosis, restoration of Nrf2 activity, and improvements in kidney histology and function with consumption of the high fiber diet was accompanied by restoration of the gut epithelial tight junction. These observations confirm the association of CKD with the impairment of the gut epithelial barrier structure and demonstrate the efficacy of the high fiber diet in minimizing this abnormality in rats with experimental CKD. In fact earlier studies by Keenan et al [20] have demonstrated the effects of the high-amylose resistant starch in improving structure and function of the gastrointestinal tract.

The precise mechanism by which HAM-RS2 retarded progression of kidney disease in the study animals is presently unknown, but a hallmark of dietary resistant starch is altered microbiota. Thus, HAM-RS2 effects may be, in part, due to a mitigating effect on uremia-induced changes in the composition and function of the intestinal microbiota. The CKD-associated changes in the intestinal microbiome can potentially accelerate CKD progression by generating harmful toxins and disrupting intestinal epithelial barrier which can intensify systemic inflammation. The complex carbohydrates in resistant starch can serve as substrates for generation of SCFA [21], [22] by symbiotic microbes [23] whose population is markedly diminished in patients with chronic renal failure [5]. As a main source of nutrients for colonocytes and T-reg cells, increased production of SCFA with a diet rich in fermentable fiber can potentially enhance the integrity of the intestinal epithelial barrier structure and function and attenuate local and systemic inflammation and hence CKD progression. In addition, the rise in population of SCFA-forming bacteria may attenuate expansion of the bacterial species that possess urease, and as such can diminish generation of NH_3_ and NH_4_OH which damage epithelial barrier in CKD. Moreover, by reacting with NH_3_ and NH_4_OH, SCFA can prevent their ability to damage the epithelial tight junction and raise their fecal excretion. Finally, by lowering the luminal pH, increased production of SCFA can reduce production of indoxyl sulfate and P-cresole sulfate which are among the major microbial derived pro-inflammatory and pro-oxidant uremic toxins. This assumption is based on earlier studies that have shown increased production of these toxic compounds at higher pH and their reduced generation at lower pH [24], [25]. The assumption that consumption of the resistant starch may have resulted in increased production of SCFAs in our CKD rats is based on earlier studies reported by members of this team in rodents fed identical high amylose starch diets [26]–[28]. Likewise dietary fiber has been shown to profoundly alter the structure and function of the intestinal microbiota in humans and experimental animals [29]. None the less since the effect of dietary resistant starch supplementation on the intestinal microbiota and SCFA production in CKD has not been previously investigated the authors plan to explore these issues in their future studies.

The fecal water content in the CKD rats fed HAM-RS2 diet was nearly four-fold higher than that found in the low-fiber CKD or healthy control rats consuming chow diet. Constipation and fluid overload are common problems in patients with advanced CKD [30]. By trapping water in the intestinal lumen, dietary fibers can safely prevent constipation. In addition, by raising fecal fluid content, these compounds can attenuate fluid overload in patients with end-stage renal disease. Likewise, by trapping ammonia, resistant starch has been shown to raise fecal ammonium [31]–[33] and thus attenuate accumulation of nitrogenous waste products in patients and animals with CKD. This phenomenon could, in part, account for the significant reduction of serum urea concentration in our CKD rats fed HAM-RS2 diet. Another mechanism by which HAM-RS2 improves kidney function may be through sustained elevation of the serum levels of the gut peptide GLP-1 [34], [35]. GLP-1 agonists have been shown to have therapeutic potential in nephropathy [36]. The salutary effect of a high fermentable fiber diet against CKD progression shown here is consistent with its well-known protective effect against various other chronic diseases, including diabetes, cardiovascular disease, colon cancer, and obesity [37].

In conclusion, consumption of high amylose maize resistant starch ameliorated inflammation and oxidative stress, reduced severity of renal injury and dysfunction, as well as increased fecal water content in rats with chronic interstitial nephropathy. Future clinical studies are needed to test efficacy of high resistant starch diet in patients with chronic kidney disease.
